# Tobacco retailer density and rurality across four US states: California, Connecticut, North Carolina, and Ohio

**DOI:** 10.1111/jrh.70073

**Published:** 2025-08-15

**Authors:** Emerson Webb, Peter F. Craigmile, Meghan E. Morean, Grace Kong, Joseph G. L. Lee, Ryan J. Martin, Jessica Barrington‐Trimis, Rui Qiang, Vitoria Borges Spinola, Megan E. Roberts

**Affiliations:** ^1^ Department of Statistics The Ohio State University Columbus Ohio USA; ^2^ Department of Mathematics and Statistics Hunter College, CUNY New York New York USA; ^3^ Department of Psychiatry Yale University School of Medicine New Haven Connecticut USA; ^4^ Division of Public Health Sciences Wake Forest University School of Medicine Winston‐Salem North Carolina USA; ^5^ College of Health and Human Performance East Carolina University Greenville North Carolina USA; ^6^ Keck School of Medicine University of Southern California Los Angeles California USA; ^7^ College of Public Health Ohio State University Columbus Ohio USA

**Keywords:** rural health, tobacco retailer density, tobacco retailer environment

## Abstract

**Purpose:**

Research has demonstrated many types of disparities in tobacco retailer density (TRD), but these analyses often fail to explore rural disparities. Given the substantial burden of rural tobacco use in the USA, this is a critical gap. The purpose of the present study was to estimate rural disparities in TRD across four US states.

**Methods:**

For the states of California, Connecticut, North Carolina, and Ohio, we used spatial statistical methods to model per capita TRD at the census tract level. Rurality was defined by the US Department of Agriculture Rural‐Uran Commuting Area (RUCA) codes and categorized into Metropolitan, Micropolitan, Small Town, and Rural.

**Findings:**

Tobacco retailer count was highest in California (22,533), but TRD was highest in Connecticut (1.23 retailers per 1000 residents). In models for California, North Carolina, and Ohio (but not Connecticut), there was an association between rurality and TRD, such that rural census tracts had greater TRD than metropolitan census tracts. Micropolitan and small town (vs. metropolitan) census tracts also had greater TRD, although the association was not as strong. Models further showed associations between TRD and census tract poverty, racial and ethnic composition, and Appalachian designation.

**Conclusions:**

Although there are notable state‐level differences, TRD is clearly associated with rurality. Given the literature on the impacts of living in tobacco‐retailer‐dense areas, rural disparities in TRD likely contribute to rural disparities in tobacco use. There is a need for further policies in rural areas of the USA that address the tobacco retailer environment.

## INTRODUCTION

The number of tobacco retailers in the US poses a critical threat to public health. The term “tobacco retailer” refers to any type of physical retail location that sells tobacco, including convenience stores, dollar stores, and tobacco shops. In the USA, it is estimated that there are over 1.22 tobacco retailers per 1000 persons.^1^ A strong body of literature indicates that greater tobacco retailer density (TRD) enables easier access to tobacco products as well as exposure to tobacco marketing.^2^, ^3^, ^4^, ^5^, ^6^ Further, youth and adults living in areas with a comparatively high (vs. low) TRD have a higher likelihood of tobacco use^6^, ^7^, ^8^, ^9^, ^10^, ^11^ and a lower likelihood of cessation success.^12^, ^13^, ^14^, ^15^, ^16^

The harmful impact of tobacco retailers not only contributes to overall population‐level tobacco use but also contributes to tobacco‐use disparities. The most robust effect in the literature is for poverty: TRD is greater in places with higher poverty.^1^, ^17^, ^18^, ^19^, ^20^, ^21^ Another commonly observed effect is for race and ethnicity: TRD is often greater in African American and Hispanic (vs. non‐Hispanic White) neighborhoods.^1^, ^17^, ^18^, ^19^, ^20^, ^21^, ^22^, ^23^, ^24^ In addition, studies have indicated that place‐based disparities in retailer density (across neighborhood income, race, and ethnicity) are directly associated with place‐based disparities in tobacco use.^25^, ^26^

Throughout this important work on retailer density disparities, ^1^, ^17^, ^18^, ^19^, ^20^, ^21^, ^22^, ^23^, ^24^, ^25^, ^26^ one place‐based characteristic that is often overlooked is rurality. Nevertheless, rural tobacco use disparities are a major public health concern. Numerous studies have documented disparities in rural tobacco use, particularly for cigarettes and smokeless tobacco.^27^, ^28^, ^29^, ^30^ One recent national survey indicated that the prevalence of using any tobacco product was 1.5 times higher for adults living in rural than in urban areas (27.3% and 17.7% prevalence, respectively).^31^ Similarly, youth use of tobacco is higher for those living in rural versus urban areas.^32^, ^33^, ^34^ Such disparities in tobacco use are concerning, as they directly contribute to disparities in rural health.^35^

The few studies that have examined the association between rurality and TRD have been mixed. One national study found that per capita TRD was higher in urban areas, compared with rural areas, in adjusted models.^36^ Another found that there is little association between county‐level retailer density and tobacco use in rural counties.^7^ A limitation to these studies, however, was that their measures likely overclassified “rural,” as they included micropolitan (i.e., suburban) areas in their rural grouping. In contrast, one study in Ohio found that per capita TRD was higher in rural areas compared with suburban areas,^37^ and one national study found that per capita TRD was higher in the combined category of small town and isolated rural compared with urban areas.^1^ Given the conflicting findings (greater density for urban areas^36^ vs. greater density for rural areas^1^), additional research looking across states is needed. Continued investigations are also necessary to determine whether a rural effect for TRD exists in varied rural contexts. For example, many rural Appalachian regions have a history of tobacco growing and cultures that are relatively favorable toward tobacco use.^30^, ^38^, ^39^ It is unclear whether other, non‐Appalachian rural areas, with different histories and cultures, equally experience elevated TRD. Greater sensitivity in geographic analyses is also warranted, including an examination of degrees of rurality.

The purpose of this study was to estimate rural disparities in TRD across different US contexts. We selected four states for this purpose: California, Connecticut, North Carolina, and Ohio. These states were selected for their varied cultural and policy landscapes; further, as each state is located within a different one of the four US Census Regions (West, Northeast, South, and Midwest), it also allowed us to explore TRD across varied geographic contexts. Our primary hypothesis was that density would be greater in rural (vs. urban) areas. We also hypothesized that, consistent with the extant literature, TRD would be greater in areas with a high prevalence of poverty and areas with a high prevalence of racially or ethnically minoritized individuals. In exploratory analyses, we also investigated the role of designation as part of the US Appalachian region.

## Methods

### Measures

#### Tobacco retailers

There is no national database of tobacco retailers in the USA, and tobacco retailer licensing is handled at the state level (if at all). Therefore, for each of our four states (California, Connecticut, North Carolina, and Ohio), we used different methods to generate a list of tobacco retailers. All data were obtained during the period 2022–2023.

In California, a license is required to sell any type of tobacco product. Names and addresses of licensed tobacco product retailers were downloaded from the California Department of Tax and Fee Administration.^40^ Tobacco distributors and wholesalers were excluded from our list.

In Connecticut, a license is only required for the sale of cigarettes and e‐cigarettes. We obtained a list of cigarette retailers from the Connecticut Department of Revenue Services^41^ and a list of e‐cigarette retailers from the Connecticut Department of Consumer Protection.^42^ For both cigarettes and e‐cigarettes, license information did not always provide the physical location of the retail store. These omissions were resolved with online searches according to the business name and/or LCC. For cigars and hookah, we conducted online searches employing methods described by Kates et al.^43^ for searching internet directories.

In North Carolina, there is no state tobacco retailer licensing. Therefore, we used protocols developed and validated in North Carolina.^44^, ^45^ Briefly, we aggregated lists of likely tobacco retailers from DataAxle, a business listing service, with off‐premises beer and wine permits from the NC Alcohol Beverage Control Commission, underground storage tank permits from the NC Department of Environmental Quality (needed by gas stations for storing gasoline), and a manual search of Google Maps for vape shops in each NC county.

In Ohio, at the time of data collection, only the cigarette license information was available. We obtained the names and addresses of all retailers with active state cigarette licenses from Ohio's county auditor offices. To collect information on hookah cafés and vape shops that did not have a state cigarette license, we employed methods described by Kates et al.^43^ for searching internet directories.

We geocoded the longitude‐latitude coordinates for each retailer address using the tidygeocoder R package.^46^ We subsequently used web searches (e.g., Google maps) in which the coordinates were used to carefully verify retailers, combine/remove duplicates, and remove purely online retailers or distributors. We independently rechecked a minimum of 10% of the retailers in each state list for accuracy.

#### Tract‐level characteristics

For sociodemographic characteristics, census‐tract‐level information about race/ethnicity, poverty, age, and population size was obtained from the 2021 American Community Survey 5‐year estimates.^47^ We considered the following sociodemographic variables for inclusion in our analyses: percentage of population below the federal poverty level, percentage identifying as non‐Hispanic Asian, percentage identifying as non‐Hispanic African American/Black, percentage identifying as Hispanic, and percentage identifying as non‐Hispanic White.

For our measure of rurality, we used Rural‐Urban Commuting Area (RUCA) 2010 classifications.^48^ We collapsed the 11 RUCA categories (see Table S1) into four broader levels: categories 1–3 as “Metropolitan”, categories 4–6 as “Micropolitan”, categories 7–9 as “Small Town”, and category 10 as “Rural.” This represents the Rural Health Research Center RUCA classification scheme A.^49^

For our measure of Appalachia, we used the official designations by the Appalachian Regional Commission (ARC).^50^ ARC designations comprise 423 counties in 13 states, including 32 counties in North Carolina and 31 counties in Ohio. We created indicator variables for the tracts in these two states to denote whether each tract was Appalachian (no/yes).

#### Geospatial shape files

We obtained TIGER shape files for census tracts in 2021 in California, Connecticut, North Carolina, and Ohio from the US Census Bureau.^51^ Guarding against modeling tracts with very low populations, we restricted our analysis to tracts with a minimum population of 100. This removed 12 census tracts (0.13%) from California and 2 census tracts (0.08%) from North Carolina. No census tracts were removed from Connecticut or Ohio.

### Statistical analyses

Analyses were carried out in R,^52^ using the sp, sf, MASS, and SPAM R libraries. For all analyses, TRD was calculated as the number of tobacco retailers per 1000 people for each census tract.

We began with mapping, descriptive statistics, and exploratory analyses to characterize the data. In addition to producing maps of the TRD, sociodemographic variables, and RUCA classification by census tract, we used the median and interquartile range (IQR) as a summary of the TRD distributions. Graphically and numerically, we explored the TRD on the original and log scales and how they related to the sociodemographic variables, RUCA classification, and Appalachian classification; the latter was just for the states served by ARC, North Carolina, and Ohio. These exploratory analyses included estimating marginal linear relationships between the log TRD and the continuous demographic variables by calculating the correlation coefficient (% below poverty, % African American, % White, % Hispanic, and % Asian). For the purposes of only this correlation calculation, we add an offset value of 0.01 before calculating the log transformation to guard against taking a log of zero.

Next, for each state, we fit negative binomial models to evaluate the association between TRD and the sociodemographic variables. The negative binomial model accounts for overdispersion in count data. Overdispersion occurs when the variance of the counts is more than would be hypothesized in a given statistical model. We adopted the approach of Adibe et al.^37^ in which the spatial effects between different census tracts are accounted for in a generalized estimating equation framework, which allows us to calculate the covariance between regression effects, suitably accounting for possible residual spatial random effects (see Supporting Information Material for more details on the model). We do this because even once we account for covariate effects, the TRDs in tracts closer together are more similar than the TRDs for tracts further apart (i.e., they are autocorrelated), which violates the independence assumption of standard statistical tests, and can underestimate standard errors. In this study, we fit separate models to the data from each state, accounting for residual spatial dependence between the census tracts in each state. To choose between various statistical models used to estimate the TRD in each state, we looked at the marginal and joint significance of terms in each model. We considered possible interactions between the RUCA classification and the sociodemographic variables in our statistical models.

Finally, for North Carolina and Ohio, we fit an additional model to each state that included the Appalachian indicator as a covariate. We examined whether there were any interactions between this Appalachian variable, the sociodemographic variables, and the RUCA classification.

We checked the fit of our models using deviance residual plots and tested for residual spatial dependence among the census tracts using Moran's *I* test statistic (e.g., Waller and Gotway^53^).

## RESULTS

### Descriptive statistics

**FIGURE 1:**
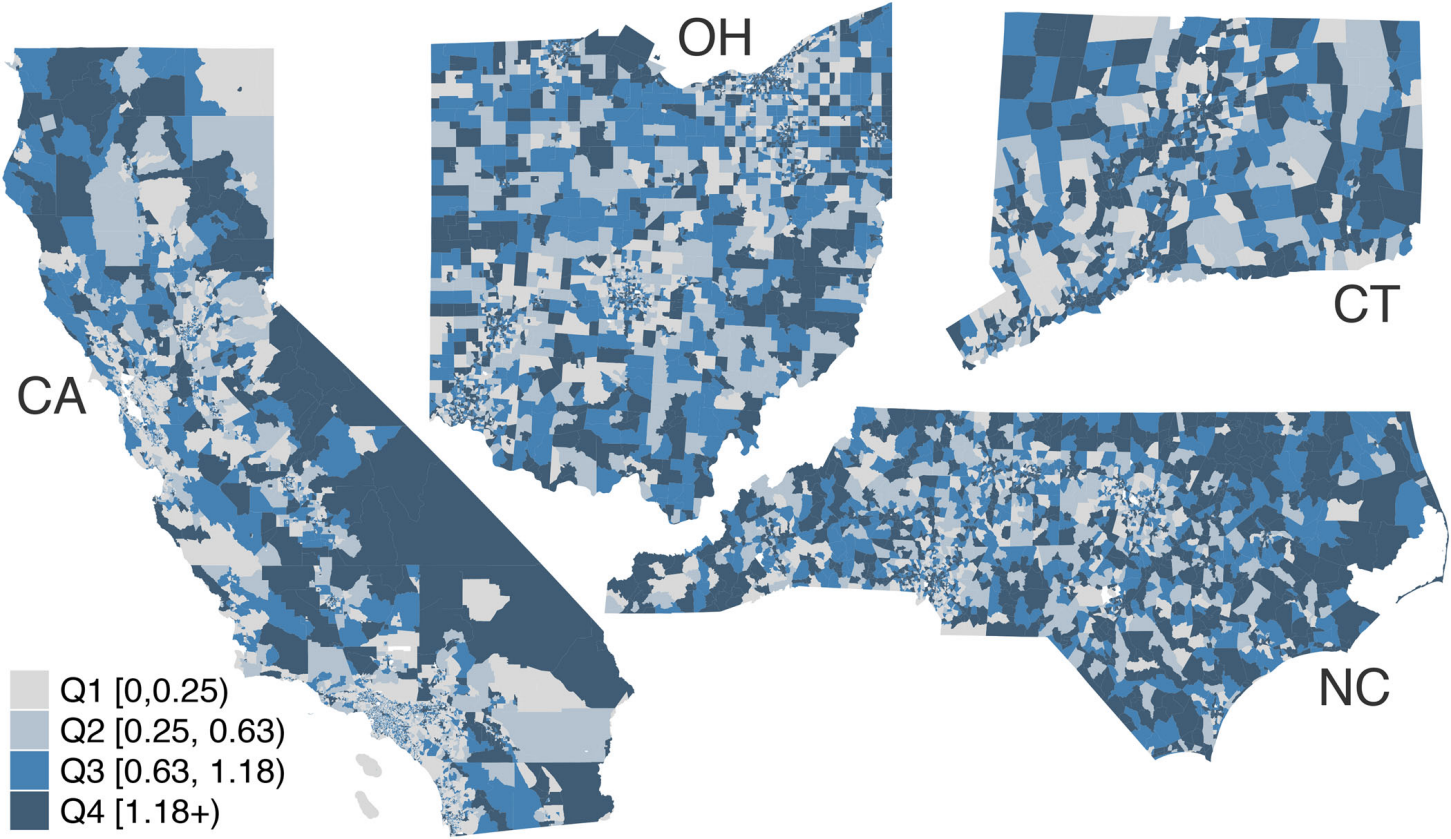
Tobacco retailer density (TRD) at the census tract level in California, Connecticut, North Carolina, and Ohio. For illustrative purposes, TRD was aggregated (over all four states) and is displayed in quantiles, where darker colors indicate greater TRD. The relative size of each state to the others is not to scale.

Our final list of tobacco retailers contained 22,533 in California, 4430 in Connecticut, 11,776 in North Carolina, and 11,167 in Ohio (Table 1). In aggregate, TRD was 0.57 in California, 1.23 in Connecticut, 1.14 in North Carolina, and 0.95 in Ohio. Thus, California had the lowest average TRD, with 0.57 tobacco retailers per 1000 people, and Connecticut had the highest average TRD, with 1.23 tobacco retailers per 1000 people (twice the density of California). Figure 1 displays maps for the four states of the geographic distribution of TRD at the census tract level.

**TABLE 1:** Descriptive statistics for California, Connecticut, North Carolina, and Ohio, including number of census tracts, median tobacco retailer density (TRD) overall and by level of rurality, and correlations between TRD, rurality, and sociodemographic characteristics.

	California		Connecticut		North Carolina		Ohio	
**# Census tracts**	9047		872		2642		3150	
**# Tobacco retailers**	22,533		4430		11,776		11,167	
**Mean TRD**	0.57		1.23		1.14		0.95	

Median TRD	%	Median (IQR)	%	Median (IQR)	%	Median (IQR)	%	Median (IQR)
**All tracts**		0.48 (0.69)		1.05 (1.38)		0.95 (1.27)		0.85 (1.02)
**Rurality (RUCA)**								
Metro	93.2	0.46 (0.67)	95.3	1.05 (1.41)	74.8	0.83 (1.16)	79.6	0.81 (1.00)
Micro	4.1	0.67 (0.86)	3.1	1.08 (0.98)	15.7	1.39 (1.55)	14.3	0.98 (0.98)
Small Town	1.1	1.01 (1.03)	0.0	– (–)	5.4	1.27 (1.89)	4.3	1.11 (1.18)
Rural	1.6	1.09 (1.23)	1.6	1.03 (0.71)	4.1	1.47 (1.65)	1.8	1.04 (0.94)

Correlation with log TRD**	Corr (*p*‐value)	Corr (*p*‐value)	Corr (*p*‐value)	Corr (*p*‐value)
% Below Poverty	0.17 (<0.001)	0.26 (<0.001)	0.27 (<0.001)	0.20 (<0.001)
% White	−0.06 (<0.001)	−0.26 (<0.001)	−0.16 (<0.001)	−0.06 (<0.001)
% African American	0.01 (0.325)	0.16 (<0.001)	0.18 (<0.001)	0.05 (0.005)
% Hispanic	0.14 (<0.001)	0.28 (<0.001)	0.08 (<0.001)	0.10 (<0.001)
% Asian	−0.13 (<0.001)	−0.04 (0.258)	−0.14 (<0.001)	−0.10 (<0.001)

*Note*: Tobacco retailer density (TRD) is defined as retailers per 1000 residents and is calculated for this table using the median and interquartile range (IQR), over all tracts. The number and percentage of tracts by Rural‐Urban Commuting Area (RUCA) classification are also presented. The bottom panel tabulates the correlations between the log TRD and other sociodemographic characteristics. The *p*‐values in parentheses after each correlation coefficient follow from tests of whether each population correlation coefficient differs from zero. Effects in bold are significantly different from zero, with significance levels ranging from *p* < 0.01 to *p* < 0.001.

Abbreviations: Metro: metropolitan; micro, micropolitan.

**FIGURE 2:**
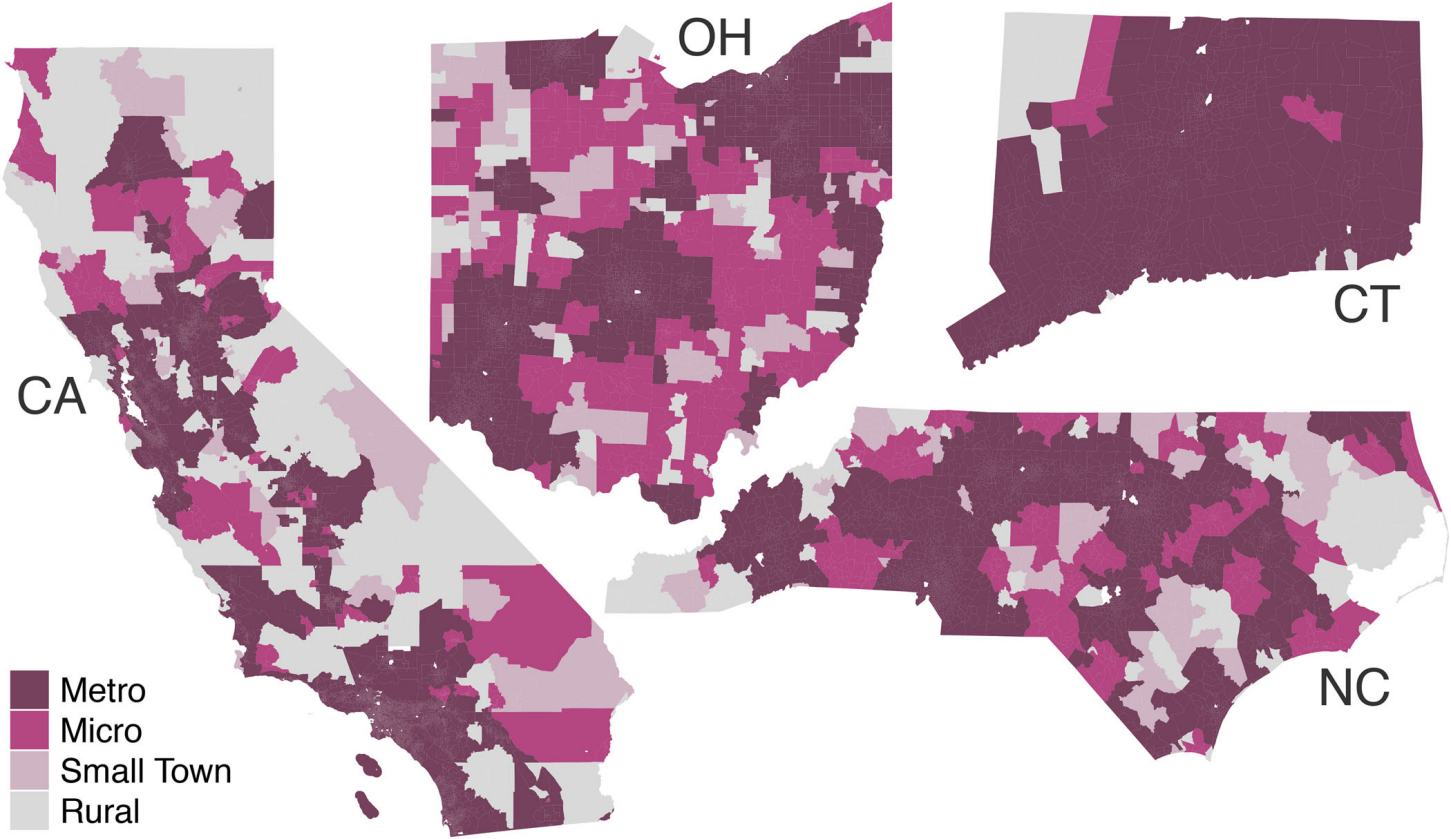
The spatial distribution of Rural‐Urban Commuting Area (RUCA) classifications across California, Connecticut, North Carolina, and Ohio.

**FIGURE 3:**
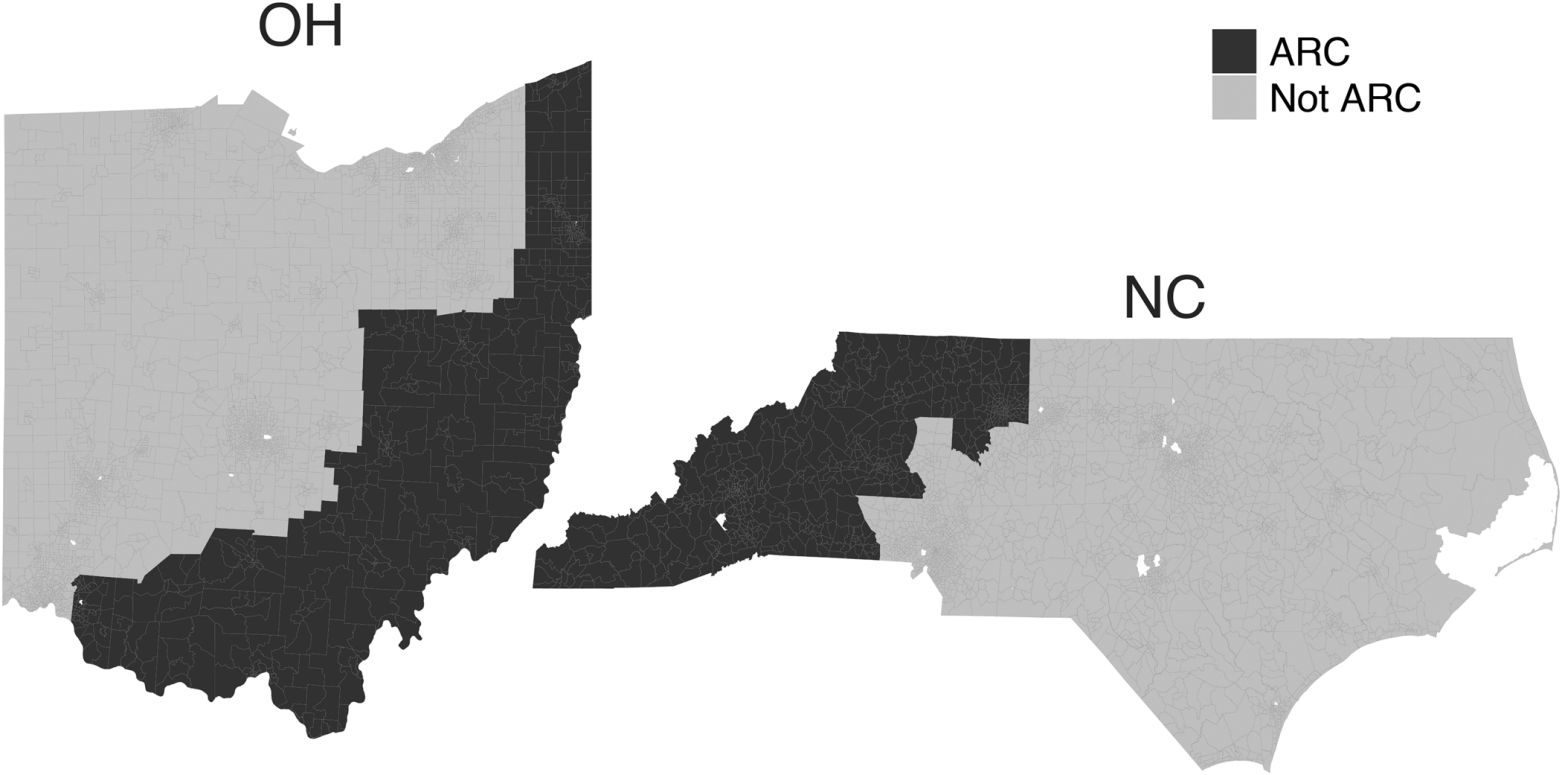
Classifications of Appalachian census tracts, based on designations by the Appalachian Regional Commission.

Figure 2 shows the spatial distribution of RUCA classifications across each state. California was characterized by some large pockets of metropolitan areas, but tracts tended to be more rural in the north, east, and southeast regions. Connecticut was largely metropolitan, except for a pocket of micropolitan and rural areas to the northwest. In North Carolina, distinct Rural pockets were present in the southwest, northwest, and eastern regions. For Ohio, the northwestern and southeastern regions were generally micropolitan or small towns, with some pockets of rural. Figure 3 shows the distribution of tracts with Appalachian status; as a visual comparison between Figures 2 and 3 makes clear, not all rural areas in the two states are Appalachian, and not all Appalachian areas in the states are rural.

The first row of Table 1 presents the number of census tracts by state, which, unsurprisingly, was positively associated with the population of each state. Connecticut had the smallest number of tracts (872), and California had the most tracts (9047). Table 1 also summarizes the distribution of TRD using the median TRD as a measure of center and the interquartile range (IQR) as a measure of spread. California had the smallest median TRD of the four states (0.48 tobacco retailers per thousand people), and Connecticut had the largest (1.38 per thousand). Next, Table 1 quantifies the state‐level variations observed in our RUCA maps. North Carolina and Ohio were comprised of more small town, rural, and micropolitan areas (and fewer metropolitan areas), compared with California and Connecticut.

Descriptive data also showed that the distribution of median TRD varied by rurality. In California, North Carolina, and Ohio, micropolitan, small town, and rural tracts tended to have higher median TRD as compared with metropolitan tracts; however, the magnitude of the differences varied by state. There was little evidence of a difference in the median TRD by RUCA classification in Connecticut. Correlations, which did not account for spatial dependence, showed that the log TRD was positively associated with tract‐level % poverty, % Hispanic, and % White (Table 1). For most states, there was also a significant association between log TRD and % Asian and % African American. We also note that while some correlations were not different from zero according to a hypothesis test, the sign of the estimated correlation values was consistent throughout.

### Spatial analysis results

Model 1 estimates (accounting for spatial dependence) the TRD in the census tracts for each state using the percentage below poverty, percentage African American, percentage Hispanic, percentage Asian, and RUCA variables (see Table 2). We excluded the percentage White variable from the final models as it was highly collinear with other demographic variables. No interactions were significant in the models involving these covariates.

**TABLE 2:** Parameter estimates (standard errors) for the marginal models, fit to each state, relating tobacco retailer density (TRD) to rurality and sociodemographic characteristics.

	California	Connecticut	North Carolina	Ohio
	Estimate (SE)	Estimate (SE)	Estimate (SE)	Estimate (SE)
**Model 1**				
Intercept	**−0.662 (0.042)**	**−0.161 (0.068)**	**−0.318 (0.047)**	**−0.325 (0.033)**
RUCA (vs. metro)				
Micro	**0.226 (0.084)**	−0.022 (0.224)	**0.336 (0.063)**	**0.147 (0.053)**
Small town	**0.428 (0.157)**	N/A	**0.330 (0.099)**	**0.291 (0.089)**
Rural	**0.625 (0.106)**	−0.015 (0.319)	**0.384 (0.119)**	**0.322 (0.117)**
% Below poverty	**0.190 (0.015)**	**0.155 (0.043)**	**0.212 (0.022)**	**0.183 (0.016)**
% African American	**−0.038 (0.019)**	0.027 (0.028)	**0.041 (0.012)**	−0.012 (0.010)
% Hispanic	−0.005 (0.007)	**0.085 (0.027)**	0.021 (0.023)	**0.072 (0.030)**
% Asian	**−0.072 (0.011)**	0.064 (0.064)	**−0.138 (0.045)**	**−0.168 (0.045)**
**Model 2**				
Intercept			**−0.386 (0.052)**	**−0.343 (0.034)**
RUCA (vs. metro)				
Micro			**0.345 (0.063)**	**0.126 (0.054)**
Small town			**0.327 (0.098)**	**0.270 (0.089)**
Rural			**0.360 (0.118)**	**0.300 (0.117)**
% Below poverty			**0.224 (0.024)**	**0.175 (0.016)**
% African American			**0.048 (0.012)**	−0.006 (0.010)
% Hispanic*			0.027 (0.023)	**0.084 (0.030)**
% Asian			**−0.123 (0.045)**	**−0.155 (0.045)**
Appalachia			**0.281 (0.097)**	**0.115 (0.053)**
% Below poverty x Appalachia interaction			**−0.011 (0.005)**	

*Note*: Models are relating tobacco retailer density (TRD) to sociodemographic variables, while accounting for spatial dependence. Model 1 is fit to all four states, while Model 2 is fit only to NC and OH, given its focus on Appalachia. For the sociodemographic variables (%below poverty, % African American, % Hispanic, and % Asian), the table summarizes the change in the mean log TRD for a 10% increase of each covariate, along with the standard error in parentheses. Rurality was measured with Rural‐Urban Commuting Area (RUCA) codes. For the RUCA, the table summarizes the change in the mean log TRD comparing each non‐metropolitan tract to metropolitan tracts (the baseline).

Effects in bold are significantly different from zero, with a threshold for statistical significance of *p <* 0.05.

Abbreviations: Metro, metropolitan; Micro, micropolitan.

Model results showed that micropolitan, small towns, and rural tracts had significantly higher TRDs (as compared with metropolitan tracts) in California, North Carolina, and Ohio. The strength of the association did vary, however, over the three states. For example, in California, we estimated a TRD 1.87 times higher for rural tracts compared with metropolitan tracts, whereas for North Carolina and Ohio, the estimated TRD was 1.47 and 1.38, respectively, times higher for rural tracts compared with metropolitan ones. In Connecticut, there were no significant differences among the TRD by rurality.

**FIGURE 4:**
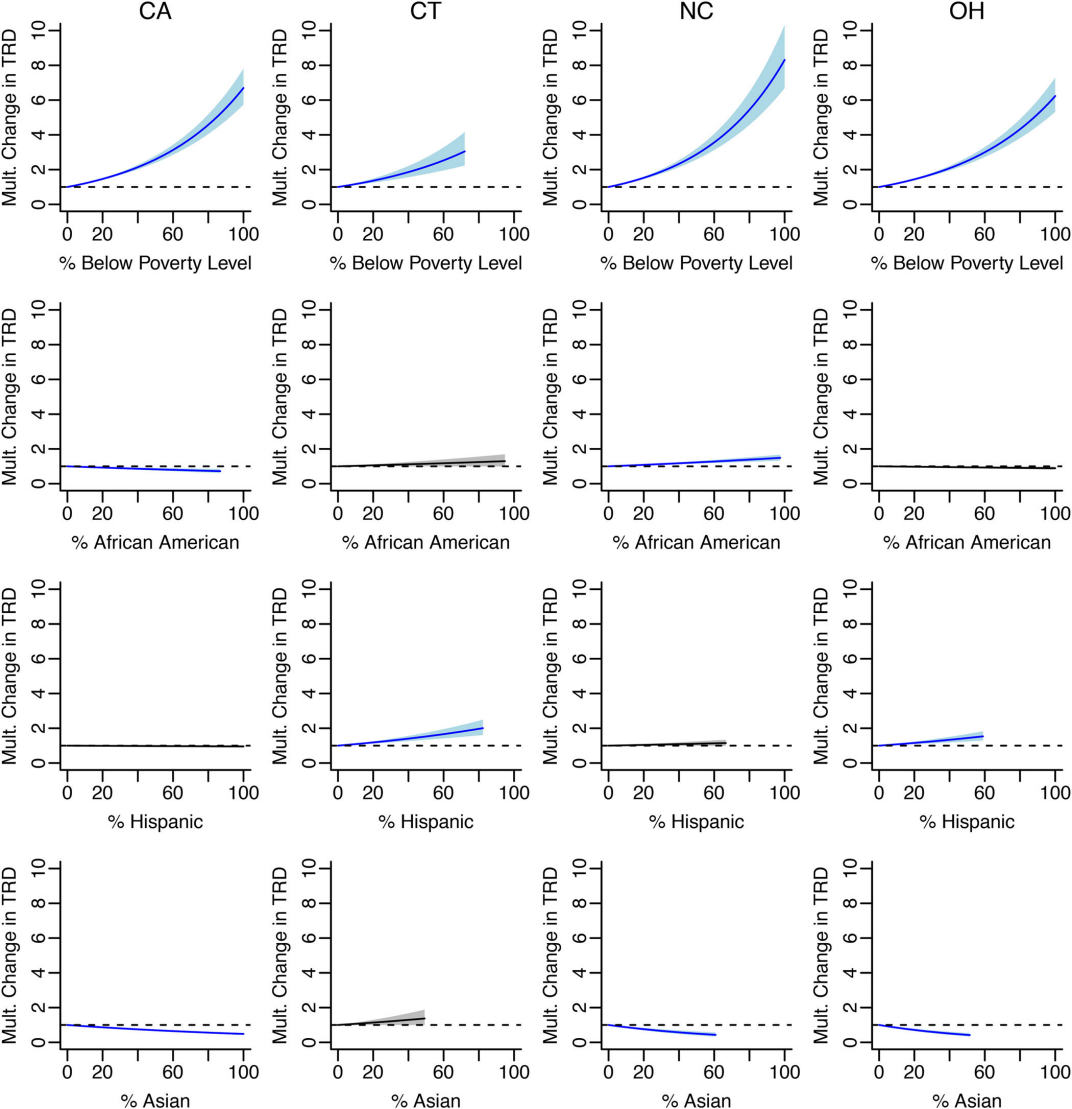
Multiplicative change in tobacco retailer density (TRD), holding each sociodemographic variable independent of the others. Columns denote each state, and rows denote different sociodemographic variables. In each panel, the solid line is the estimated multiplicative change in the TRD as the sociodemographic variable increases, and the shared region denotes a pointwise 95% confidence interval for this change. The horizontal dashed line indicates a value of one (i.e., no change in the multiplicative change in the TRD). Blue lines indicate significant changes. Note: Effects are only plotted for the ranges observed in the datasets for each state. For this reason, some lines do not extend to 100% on the *x*‐axis (e.g., in CT, there were no tracts with >80% poverty).

Table 2 and Figure 4 demonstrate that poverty was associated with the largest multiplicative increase in TRD. Comparing between states, the strongest association between poverty and TRD was in North Carolina, followed by California, then Ohio, and then Connecticut. Specifically, TRD increased by a factor of 1.24 in North Carolina, 1.21 in California, 1.20 in Ohio, and 1.17 in Connecticut for every 10% increase in the % below poverty.

For tracts with a larger percentage of African American individuals, we observed a significant difference in the TRD within California and North Carolina. Specifically, the estimated TRD changes by a factor of 0.96 in California and 1.04 in North Carolina for every 10% increase in % African Americans in tracts. The association between the percentage of African American individuals and TRD in Connecticut and Ohio was not significant. On the other hand, for tracts with a larger percentage of Hispanic individuals, we observed significantly lower TRD within Connecticut and Ohio. Specifically, the estimated TRD changed by a factor of 1.09 for Connecticut and 1.07 for Ohio for every 10% increase in the % Hispanic; there were no significant associations in California or North Carolina. For tracts with a larger percentage of Asian individuals, we observed significantly lower TRD for California, North Carolina, and Ohio. Specifically, the estimated TRD changed by factors of 0.93, 0.87, and 0.85, respectively, for every 10% increase in % Asians; there was no significant association for the percentage of Asian individuals in Connecticut.

### Including Appalachian status

In models for North Carolina and Ohio that added an indicator variable for Appalachia, the relative magnitude, direction, and significance of coefficients already present in Model 1 did not appreciably change (see Table 2, Model 2). In Ohio, the Appalachian variable was significant, estimating that TRD was 1.22 times higher for Appalachian tracts over non‐Appalachian tracts. No interactions with the Appalachian variable were significant in the Ohio model.

In North Carolina, the association between Appalachia and TRD was only significant when the interaction with poverty was included. As in Ohio, TRD was higher among Appalachian (vs. non‐Appalachian) tracts overall. However, the association between poverty and TRD was more pronounced for non‐Appalachian (vs. Appalachian) tracts, particularly when a very low percentage of the population was living under the poverty line. For example, in tracts with 12.4% of people below poverty (the median value), the estimated TRD was 1.31 times higher in Appalachian tracts compared with non‐Appalachian tracts. In tracts with 20% and 34.2% of people below poverty (the 0.75 and 0.95 quantiles), TRD was not significantly different between Appalachian and non‐Appalachian tracts.

### Further exploring rural effects

As shown in Table 3, we further investigated the rural effects found in Model 1 by comparing each of the four‐level rural classifications to one another. Replicating Model 1 results, we found that, for California, North Carolina, and Ohio, metropolitan tracts had lower TRD than micropolitan, small town, and rural tracts. For California, North Carolina, and Ohio, there was no difference in the TRD between small town and micropolitan tracts, or between rural and small‐town tracts. For California, the estimated TRD was 1.48 times higher for rural tracts compared with micropolitan tracts. For North Carolina and Ohio, there was no difference in the TRD between rural and micropolitan tracts.

**TABLE 3:** From Model 1, the estimated tobacco retailer density ratios, fit to each state, comparing each rural classification to one another.

	California	Connecticut	North Carolina	Ohio
	TRD ratio (95% CI)	TRD ratio (95% CI)	TRD ratio (95% CI)	TRD ratio (95% CI)
Micro vs. metro	**1.254 (1.006, 1.567)**	0.978 (0.572, 1.673)	**1.399** (**1.236, 1.584)**	**1.159** (**1.006, 1.334)**
Small town vs. metro	**1.535 (1.015, 2.319)**	–	**1.391** (**1.146, 1.688)**	**1.338** (**1.059, 1.691)**
Rural vs. metro	**1.867 (1.410, 2.473)**	0.985 (0.459, 2.114)	**1.468** (**1.164, 1.853)**	**1.380** (**1.013, 1.880)**
Small town vs. micro	1.222 (0.773, 1.925)	–	0.994 (0.804, 1.229)	1.155 (0.899, 1.483)
Rural vs. micro	**1.489 (1.067, 2.078)**	1.007 (0.404, 2.510)	1.050 (0.820, 1.343)	1.191 (0.864, 1.641)
Rural vs. small town	1.218 (0.760, 1.946)	–	1.056 (0.797, 1.399)	1.031 (0.715, 1.487)

*Note*: Rurality was measured with Rural‐Urban Commuting Area (RUCA) codes. Values in parentheses denote 95% simultaneous confidence intervals for the tobacco retailer density ratios, accounting for all pairwise comparisons using the Bonferroni correction within each state.

Effects in bold are significantly different from one, with a threshold for statistical significance of *p <* 0.05.

Abbreviations: Metro, metropolitan; Micro, micropolitan.

## DISCUSSION

This study examined the TRD of four states that differed greatly in their sociodemographic and geographic characteristics. Across all four states, we found that poverty was consistently the strongest predictor of TRD: the higher the prevalence of a tract's poverty, the higher its per capita density of tobacco retailers. This finding is consistent with what has been observed in other TRD studies.^1^, ^17^, ^18^, ^19^, ^20^ A more novel finding that emerged from this study was that rurality was associated with TRD for most states. Specifically, for California, North Carolina, and Ohio, tracts that were classified as rural experienced greater TRD, compared with tracts classified as metropolitan. In fact, we observed a stepwise, or exposure‐response,^54^ association, such that TRD was modestly higher in micropolitan and small‐town areas (vs. metropolitan areas), but the greatest difference was observed for rural (vs. metropolitan) areas in the RUCA scale. This observed pattern aligns with our premise for this paper, which is that TRD is a contributing factor to the elevated prevalence of rural tobacco use.^27^, ^28^, ^29^ Previous longitudinal work would suggest that these TRD disparities are not merely a matter of tobacco retailers responding to where there is a high prevalence of tobacco use (i.e., “supply and demand”); rather, longitudinal analyses indicate that the relationship between TRD and tobacco is reciprocal, with use prevalence driving subsequent TRD, but TRD also driving increases in use prevalence.^55^

Notably, an association between rurality and TRD was not observed for Connecticut. It is possible that this null finding for Connecticut was due to its geographic layout, which has fewer rural tracts compared with our other states. Alternatively, the differences may reflect other unmeasured economic factors, such as where there has (vs. has not) been a growth of dollar stores.^56^, ^57^ It will be important for future research to further investigate this type of state‐to‐state variation and to determine what drives the association between rurality and TRD in some states but not others. Uncovering this state‐level variation is particularly important for understanding why associations between rurality and TRD are not consistently observed at the national level.^1^, ^36^ As demonstrated by the present findings, the lack of a national effect in some studies does not mean that rurality does not matter for TRD. In fact, we observed that rurality mattered for 3 out of the 4 states examined, where it showed strong associations with TRD. Thus, national effects belie important regional variations.

Our study also investigated the unique association between being part of the Appalachian region and TRD. Here, again, we uncovered important state‐level differences. Specifically, Ohio showed a main effect for an Appalachian designation, such that TRD was greater in Appalachian (vs. non‐Appalachian) areas of the state. This association between rurality and TRD appeared even in a model that accounted for other factors, such as poverty and rurality. North Carolina additionally demonstrated an interaction, whereby the difference between Appalachian versus non‐Appalachian regions was most pronounced in census tracts with very low poverty. One potential explanation for this pattern is the influence of tobacco growing in the state. For instance, Winston‐Salem, North Carolina, is an Appalachian city that experiences higher income relative to the rest of North Carolina and whose centrality to the tobacco industry likely contributes to its higher retailer density (Reynolds American, Inc., a subsidiary of British American Tobacco, maintains a presence in that city).

Finally, our study detected state‐specific disparities in terms of race and ethnicity, after accounting for poverty and geographic characteristics. For example, North Carolina had greater TRD in tracts with a high prevalence of African American individuals. In contrast, Connecticut and Ohio observed greater TRD in tracts with a high prevalence of Hispanic individuals. Thus, all states experience greater TRD for a racially or ethnically minoritized population, but the specific population impacted (e.g., African American or Hispanic) depended on the state. These findings are consistent with the existing literature on TRD, where significant associations have been found in most, but not all, studies examining race/ethnicity‐based disparities (see Kong et al.^21^ for a review). These findings again reinforce the importance of examining state‐level trends.

It is important to note the limitations of this investigation. Whereas our study used retailers per capita as a measure of density, there are other measures of density (e.g., retailers per land area) that would have likely produced different results;^58^ all are imperfect proxies for tobacco availability. Another primary limitation is that we only investigated four states, and findings may not generalize to other areas. In the USA, there is no national database of tobacco retailers. Researchers who wish to investigate national trends often rely on commerce codes, which estimate probable tobacco retailers. Although useful for large‐scale analyses, this method for estimating probable tobacco retailers sacrifices accuracy, as it can incorrectly include some retailers that are not actually selling tobacco,^1^ while also missing other retailers that are.^59^ Alternatively, we chose a method that provides greater accuracy for identifying tobacco retailer locations, at the trade‐off of a lengthier data acquisition process and a smaller database.

## CONCLUSION

This study documents rural disparities in TRD across different regions of the USA. In three out of four states examined (California, North Carolina, and Ohio, but not Connecticut), we observed that an increasing level of rurality is associated with an increasing degree of TRD. This finding is concerning, given the pronounced rural disparities in tobacco use.^27^, ^28^, ^29^, ^30^, ^31^, ^32^, ^33^, ^34^ A strong body of literature ties greater TRD to greater tobacco access and marketing exposure, as well as prospective patterns in youth initiation and population prevalence.^2^, ^3^, ^4^, ^5^, ^6^, ^7^, ^8^, ^9^, ^10^, ^58^ Thus, it is very likely that TRD contributes to rural disparities in tobacco use and tobacco‐related disease. There is a need for further tobacco control policy in rural areas of the USA to reduce and restrict TRD.

## CONFLICT OF INTEREST STATEMENT

The authors declare no conflicts of interest. Joseph G. L. Lee has a royalty interest in a retailer mapping and audit software owned and licensed by the University of North Carolina at Chapel Hill. The software was not used in this research.

## Supporting information



Supporting Information
