# Supplementary material for: Tobacco retailer density and rurality across four US states: California, Connecticut, North Carolina, and Ohio
Source: J Rural Health. 2025 Aug 15;41(3):e70073. doi: 10.1111/jrh.70073 (PMC12357071; doi:10.1111/jrh.70073)
Supplement: Supplementary file 1 — Supporting Information [file JRH-41-0-s001.pdf]

Supplemental material for "Understanding the relationship between tobacco retailer density and rurality across 4 U.S. states"

Emerson Webb, Peter F. Craigmile, Meghan E. Morean, Grace Kong, Joseph Lee, Ryan J. Martin, Jessica Barrington-Trimis, Rui Qiang, Vitoria Borges Spinola, Megan E. Roberts

Last updated April 4, 2025

In this supplemental material, we describe how we define the spatial statistical model that relates the tobacco retailer counts to covariates over the census tracts for each state. We also give an estimate of the covariance of the coefficient parameter vector using a sandwich estimator, and provide further detail of the Rural-Urban Commuting Area (RUCA) 2010 classifications.

S1 Defining the spatial model for retailer counts in each state

Let k denote the indicator of state, where $k = 1$: California, $k = 2$: Connecticut, $k = 3$: North Carolina, and $k = 4$ Ohio.

Then, in each state k suppose that there are m_k census tracts. Let Y_{ki} denote the number of tobacco retailers in census tract $i = 1, \ldots, m_k$ of state k, P_{ki} denote the number of people (in thousands) living in census tract i of state k, and let $\boldsymbol{x}_{ki}$ be a length p_k vector covariates for each census tract i in state k.

We model each state $k = 1, \ldots, 4$ separately. Our marginal model for the tobacco retailer counts in each state k assumes that the mean number of tobacco retailer counts in each

census tract i of state k, μ_{ki}, satisfies

$$\mu_{ki} \;=\; E(Y_{ki}) \;=\; P_{ki}\exp(\boldsymbol{x}_{ki}^T\boldsymbol{\beta}_k), \quad i = 1, \ldots, m_k,$$

where $\boldsymbol{\beta}_k$ is a coefficient parameter vector of length p_k. Thus the tobacco retailer density (TRD) in census tract i of state k is

$$\text{TRD}_{ki} \;=\; \frac{\mu_{ki}}{P_{ki}} \;=\; \exp\left(\boldsymbol{x}_{ki}^T\boldsymbol{\beta}_k\right), \quad i = 1, \ldots, m_k.$$

In our model we assume that the variance of the tobacco retailer counts in census tract i of k, $V_k(\mu_{ki})$, is

$$V_k(\mu_{ki}) = \text{var}(Y_{ki}) = \sigma_k^2\left[\mu_{ki} + \frac{\mu_{ki}^2}{\theta_k}\right], \quad i = 1, \ldots, m_k,$$

where $\sigma_k^2 > 0$ is a variance parameter that can be different for each state k, and $\theta_k > 0$ is an overdispersion parameter that can also vary by state. Within a state we assume that the tobacco retailer counts in each tract are spatially correlated. The covariance between the counts in tract i and i' is

$$\text{cov}(Y_{ki}, Y_{ki'}) \;=\; \sqrt{V_k(\mu_{ki})V_k(\mu_{ki'})}\,[R_k]_{ii'},$$

where $[R_k]_{ii'}$ is the (i, i') element of a $m_k \times m_k$ spatial correlation matrix $\boldsymbol{R_k}$ that corresponds to assuming a conditional autoregressive (CAR) spatial model (e.g. Banerjee et al., 2014) across the m_k census tracts in each state k. We assume that $\boldsymbol{R}_k$ is defined by

$$\boldsymbol{R}_k = (\boldsymbol{D}_k - \alpha_k\boldsymbol{W}_k)^{-1}, \tag{S1}$$

where $\boldsymbol{W}_k$ is a $m_k \times m_k$ spatial proximity matrix with (i, i') element equal to one if tract i and tract i' within state k share a border, and zero otherwise. The diagonal elements of $\boldsymbol{W}_k$ are assumed to be zero. The $m_k \times m_k$ matrix $\boldsymbol{D}_k$ is a diagonal matrix with ith diagonal element equal to the number of census tracts in state k that share a border with census tract i. In (S1), the parameter α_k denotes a spatial dependence parameter that lies between -1 and 1, and can vary by the state k. A value of $\alpha_k > 0$ for a given state indicates positive

residual spatial dependence across the tracts of a given state, once we account for possible covariate and variance effects in our statistical model.

We use a generalized estimating equation (GEE) methodology to fit our model. We first fit negative binomial generalized linear models to the tobacco retailer counts across the m_k census tracts for each state – this gives estimates of the coefficient parameter vectors $\boldsymbol{\beta}_k$ and overdispersion parameters θ_k. From the Pearson residuals we estimate the remaining parameters in the model for each state (σ_k^2 and α_k) using maximum likelihood. (See below.)

S2 Estimating the covariance matrix for the coefficient vector

Let $\boldsymbol{Y}_k = (Y_{k1}, \ldots, Y_{km_k})^T$ denote the vector of tobacco retailer counts for all census tracts in state k and $\boldsymbol{X}_k$ denote the $m_k \times p_k$ design matrix with ith row equal to the covariate vector $\boldsymbol{x}_{ki}$ for census tract i of state k. Let $\boldsymbol{G}_k$ be an $m_k \times p_k$ matrix with (i, j) element $\mu_{ki}\, [\boldsymbol{x}_{ki}]_j$, and let $\boldsymbol{J}_k = \boldsymbol{G}_k^T \boldsymbol{V}_k^{-1} \boldsymbol{G}_k$ where $\boldsymbol{V}_k = \mathrm{diag}(V_k(\mu_{ki}) : i = 1, \ldots, m_k)$ is the $m_k \times m_k$ working covariance matrix assuming independence over space for each state k.

Then, the sandwich estimator for the covariance of the estimated coefficient parameter vector $\widehat{\boldsymbol{\beta}}_k$ in state k is

$$\mathrm{cov}(\widehat{\boldsymbol{\beta}}_k) \quad = \quad \boldsymbol{J}_k^{-1} \boldsymbol{G}_k^T \boldsymbol{V}_k^{-1} \mathrm{cov}(\boldsymbol{Y}_k) \boldsymbol{V}_k^{-1} \boldsymbol{G}_k \boldsymbol{J}_k^{-1}.$$

Using maximum likelihood (ML), the parameters σ_k^2 and α_k are estimated from the Pearson residuals for all census tracts in state k. With these estimates, our estimated covariance of the estimated coefficient parameter vector $\widehat{\boldsymbol{\beta}}_k$ in state k is

$$\widehat{\mathrm{cov}}(\widehat{\boldsymbol{\beta}}_k) \quad = \quad \widehat{\sigma}_k^2\, \boldsymbol{J}_k^{-1} \boldsymbol{B}_k^T \left(\boldsymbol{D}_k - \widehat{\alpha}_k \boldsymbol{W}_k\right)^{-1} \boldsymbol{B}_k \boldsymbol{J}_k^{-1},$$

where $\boldsymbol{B}_k = \mathrm{diag}\Big(\widehat{\mu}_{ki} / \sqrt{V_k(\widehat{\mu}_{ki})} : i = 1, \ldots, m_k\Big)\, \boldsymbol{X}_k$.

S3 Rural-Urban Commuting Area (RUCA) 2010 classifications

Table S1 provides a list of the Rural-Urban Commuting Area (RUCA) 2010 classifications.

Table S1: A description of the RUCA 2010 classifications, obtained from the Economic Research Service.

Value	Description
1	Metropolitan area core: primary flow within an urbanized area (UA)
2	Metropolitan area high commuting: primary flow 30% or more to a UA
3	Metropolitan area low commuting: primary flow 10% to 30% to a UA
4	Micropolitan area core: primary flow within an Urban Cluster of 10,000 to 49,999 (large UC)
5	Micropolitan high commuting: primary flow 30% or more to a large UC
6	Micropolitan low commuting: primary flow 10% to 30% to a large UC
7	Small town core: primary flow within an Urban Cluster of 2,500 to 9,999 (small UC)
8	Small town high commuting: primary flow 30% or more to a small UC
9	Small town low commuting: primary flow 10% to 30% to a small UC
10	Rural areas: primary flow to a tract outside a UA or UC
99	Not coded: Census tract has zero population and no rural-urban identifier information

References

Banerjee, S., Carlin, B. P., and Gelfand, A. E. (2014). *Hierarchical Modeling and Analysis for Spatial Data*. Chapman and Hall/CRC, Boca Raton, FL.
